# Subclinical Hypocalcemia in Dairy Cows: An Integrative Evaluation of Blood Biomarkers, In-Line Milk Composition, and Rumination Behavior

**DOI:** 10.3390/life15121810

**Published:** 2025-11-26

**Authors:** Samanta Grigė, Akvilė Girdauskaitė, Lina Anskienė, Ieva Rodaitė, Eimantas Ginkus, Karina Džermeikaitė, Justina Krištolaitytė, Greta Šertvytytė, Gabija Lembovičiūtė, Ramūnas Antanaitis

**Affiliations:** 1 Large Animal Clinic, Veterinary Academy, Lithuanian University of Health Sciences, 44307 Kaunas, Lithuania; akvile.girdauskaite@lsmu.lt (A.G.); ieva.rodaite@lsmu.lt (I.R.); eimantas.grinkus@lsmu.lt (E.G.); karina.dzermeikaite@lsmu.lt (K.D.); justina.kristolaityte@lsmu.lt (J.K.); greta.sertvytyte@lsmu.lt (G.Š.); gabija.lemboviciute@lsmu.lt (G.L.); ramunas.antanaitis@lsmu.lt (R.A.); 2 Department of Animal Breeding, Faculty of Animal Sciences, Lithuanian University of Health Sciences, 44307 Kaunas, Lithuania; lina.anskiene@lsmu.lt

**Keywords:** innovative technologies, hypocalcemia, biomarkers, milk composition dairy cows

## Abstract

Subclinical hypocalcemia (SCH) is one of the most prevalent metabolic disorders in early-lactation dairy cows, yet its multifaceted physiological effects are often overlooked due to the absence of clinical symptoms. This study aimed to characterize SCH through an integrative assessment of blood biochemical markers, in-line milk composition, and sensor-derived behavioral traits. Seventy-five Holstein cows between 2 and 21 days in milk were classified into hypocalcemic (group 1) (Ca < 2.0 mmol/L; *n* = 20) and healthy (group 2) groups (*n* = 55). Blood samples, milk data, and rumination metrics were evaluated, and group differences were analyzed using Welch’s *t*-test and Pearson correlations. Cows with SCH exhibited significantly lower concentrations of Ca, PHOS, Mg, ALB, TP, GLUC, and Fe, indicating disruptions in mineral balance, protein metabolism, and energy status. Hepatic indicators (AST, ALT, GGT) did not differ between groups, whereas CREA was significantly lower in hypocalcemic cows, suggesting altered muscle metabolism rather than impaired renal function. Although differences in milk yield, composition, and rumination time did not reach statistical significance, hypocalcemic cows showed consistent biological tendencies toward reduced milk components and lower milk temperature. Correlation analysis revealed strong physiological linkages among Ca, ALB, P, TP, and Fe, underscoring the interconnected nature of mineral and protein metabolism in early lactation. These findings demonstrate that SCH is associated with coordinated biochemical and behavioral changes even in the absence of clinical signs. Integrating blood biomarkers with real-time sensor data provides a more comprehensive understanding of calcium-related metabolic challenges and highlights the potential of precision-livestock technologies for early detection. Future studies incorporating ionized calcium and longitudinal sampling are needed to refine diagnostic thresholds and improve predictive monitoring of SCH in dairy herds.

## 1. Introduction

Hypocalcemia in dairy cattle, commonly referred to as milk fever or periparturient hypocalcemia, is a metabolic disorder that arises around calving when the rapid increase in calcium required for colostrum and milk synthesis exceeds the cow’s ability to maintain normal blood calcium concentrations [1,2]. Subclinical hypocalcemia (SCH) is particularly widespread, affecting an estimated 40–50% of multiparous cows postpartum, while clinical manifestations occur in a much smaller proportion of animals—approximately 7% in some herds. SCH is generally defined by total blood calcium concentrations between 1.38 and 2.0 mmol/L (5.5–8.0 mg/dL) [3]. Although the transition period—defined as the three weeks before and after calving—poses the highest risk for hypocalcemia, some studies have reported the presence of subclinical hypocalcemia at various stages of lactation. While cows in early lactation generally exhibit lower serum calcium concentrations than those in later stages, the overall prevalence of SCH does not appear to differ significantly across the lactation cycle [4]. Despite its high prevalence, the vulnerability of the periparturient period and the importance of timely preventive measures are often underestimated by both farmers and practitioners [5,6].

Calcium is a key macromineral required for neuromuscular function, endocrine regulation, blood coagulation, immune responsiveness, and skeletal metabolism [7]. Maintaining calcium balance during the transition period depends on rapid endocrine adjustments, including parathyroid hormone release, vitamin D activation, and mobilization of calcium from bone reserves. When these adaptive mechanisms are insufficient, cows experience compromised smooth muscle contraction, impaired immune cell function, and broader metabolic instability—even before clinical signs become evident [8]. SCH may persist or recur later in lactation, particularly in older cows, and is frequently accompanied by alterations in serum phosphorus, albumin, total protein, and cholesterol, indicating disruptions in energy balance and liver function [4].

Economically, SCH represents a major burden to dairy systems. Its impact on milk production, fertility, and health contributes to substantial direct and indirect losses. For example, in Holstein Friesian × Zebu crossbred cows in North-West Ethiopia, an SCH prevalence of 20.3% was associated with reduced milk yield, leading to an estimated loss of 3026.25 Ethiopian Birr (approximately 69.6 USD) per cow per lactation from decreased production alone [9]. Similarly, data from Eastern European farms indicate that cows with postpartum SCH required more insemination attempts and experienced longer service periods and calving intervals. Even where milk yield differences were minimal, increased mastitis incidence and elevated somatic cell counts contributed to additional costs of approximately 54 EUR per affected cow compared with normocalcemic animals [7].

Traditional prevention strategies have focused on calcium supplementation and dietary management. However, recent research highlights the multifactorial nature of hypocalcemia, involving complex interactions among immune, endocrine, and metabolic pathways. Effective control therefore requires a multidisciplinary approach that integrates advances in nutrition, immunology, microbiology, genetics, and endocrinology [10]. Nevertheless, the consistent application of preventive strategies remains limited, emphasizing the need for better monitoring and early detection tools to reduce the health and economic consequences of hypocalcemia in dairy herds [4,6].

The rapid expansion of precision-livestock technologies offers new opportunities to address these challenges. Real-time in-line milk analyzers, rumination collars, activity sensors, and automated feeding systems now provide continuous, cow-level data on milk composition, behavior, and physiological status, capturing early deviations that may reflect underlying metabolic stress [11]. When these sensor-derived metrics are integrated with blood biomarkers of mineral balance, energy metabolism, liver function, and inflammation, they allow a more comprehensive assessment of the pathophysiology of SCH. Such integrative approaches may support the development of non-invasive indicators, enhance transition-cow management, and enable earlier and more targeted interventions [12].

This study aims to evaluate subclinical hypocalcemia in dairy cows using a combined analysis of blood biochemical markers, in-line milk composition, and behavioral metrics. By linking traditional physiological measures with high-frequency sensor data, we seek to generate a more holistic understanding of SCH and its broader implications for cow health, performance, and precision monitoring in modern dairy systems.

## 2. Materials and Methods

### 2.1. Animal Housing, Feeding and Management Conditions

The animal experiment was approved by the State Food and Veterinary Service of the Republic of Lithuania (approval No. 135834789) and conducted in accordance with national legislation and institutional ethical requirements. The study was carried out in September 2025 on a commercial dairy farm located in the central region of Europe (56°00′ N, 24°00′ E).

All rations were formulated by a licensed dairy nutritionist to meet nutrient requirements appropriate for each physiological stage, following NRC recommendations. Lactating cows received a total mixed ration consisting primarily of grain concentrate mash, which accounted for approximately 50% of the diet on a dry matter basis. The forage portion included corn silage at roughly 30%, grass silage at approximately 10%, and grass hay at about 4% of the ration. Additional fiber and energy sources such as wheat straw, lucerne hay containing 13% crude protein, sugar beet pulp silage, and compound feed were incorporated as part of the farm’s standard feeding program. All ration components were mixed daily and delivered as a total mixed ration, and cows had unrestricted access to clean drinking water.

Dry cows were fed a transition diet formulated to support metabolic stability and fetal growth, consisting of 1.2 kg per cow per day of rapeseed meal (36% crude protein), 8.0 kg per cow per day of grass silage containing 27% dry matter, 1.2 kg per cow per day of maize silage with 27% dry matter, and 7.5 kg per cow per day of wheat straw. The ration also included 0.25 kg per cow per day of a commercial dry cow mineral and vitamin supplement providing essential macro-minerals, trace elements, and fat-soluble vitamins, and approximately 4.3 kg per cow per day of water was added to the mixture to improve feed structure and reduce sorting. During the transition period, dry cows were gradually introduced to components of the lactating-cow ration to facilitate rumen adaptation and minimise metabolic disturbances after calving. Fresh drinking water was available ad libitum to all animals.

The cows were housed in a loose housing system and were fed a total mixed ration (TMR) balanced to meet their physiological requirements throughout the year. The TMR consisted of approximately 50% grain concentrate mash, 30% corn silage, 10% grass silage, and 4% grass hay. The diet was formulated to provide adequate nutrients for a 550 kg Holstein cow producing 35 kg of milk per day. Feeding took place twice daily at 06:00 and 18:00.

### 2.2. Data Collection

The cows were milked using Lely Astronaut^®^ A3 milking robots (Lely, Maassluis, The Netherlands) operating in a free-traffic system. To encourage voluntary milking, each cow received approximately 2 kg of concentrate per day while in the milking unit. Milk-related parameters—including milk protein (MP), milk fat (MF), milk yield (MY), milk lactose (LAC), fat-to-protein ratio (FPR), somatic cell count (SCC), and electrical conductivity (EC; average of all four quarters)—were automatically recorded by the Lely Astronaut^®^ A3 robots and stored in the Lely T4C management system. For this study, in-line milk data were extracted as daily averages corresponding to the day of blood sampling to ensure temporal alignment with serum biochemistry.

RT data were obtained from Lely Qwes-H/HR activity and rumination tags (Lely, Maassluis, The Netherlands), which use a neck-mounted accelerometer and jaw-movement pattern recognition to classify rumination behavior. The tags recorded rumination at 2 min intervals and transmitted data wirelessly to the barn antenna network, where they were automatically integrated into the Lely T4C management software for each cow. These devices and their firmware-based classification algorithm are routinely used in commercial herds and have been validated for monitoring rumination behavior in dairy cows.

Blood sampling was performed at 10:00 a.m., prior to the afternoon feeding. Approximately 10 mL of blood was collected from the coccygeal vein into standard evacuated red-top tubes (BD Vacutainer, Crawley, UK). Samples were transported to the Laboratory of Clinical Tests, Large Animal Clinic, Veterinary Academy, Lithuanian University of Health Sciences, for biochemical analysis.

Serum was analyzed using a Hitachi 705 analyzer (Hitachi, Tokyo, Japan) with DiaSys reagents (Diagnostic Systems GmbH, Dusseldorf, Germany). Non-esterified fatty acids (NEFA) were determined using an Rx Daytona automated wet chemistry analyzer (Randox Laboratories Ltd., London, UK). Calibration and internal quality control for all serum biochemical measurements were carried out according to the manufacturers’ specifications. The Hitachi 705 analyzer was calibrated using DiaSys multipoint calibration standards with built-in reagent lot–specific factors.

The following biochemical parameters were measured:

Albumin (ALB), aspartate aminotransferase (AST), gamma-glutamyltransferase (GGT), alanine aminotransferase (ALT), calcium (Ca), creatinine (CREA), C-reactive protein (CRP), iron (Fe), glucose (GLUC), lactate dehydrogenase (LDH), magnesium (Mg), phosphorus (PHOS), total protein (TP), triglycerides (TRIG), urea (UREA), sodium (Na), potassium (K), and chloride (Cl). Table 1 summarizes all investigated parameters.

### 2.3. Grouping

From a total herd of 2300 Holstein cows, seventy-five early lactation animals (30 primiparous and 45 multiparous) between 2 and 21 days in milk were selected for detailed monitoring. Cows were to be excluded if they exhibited clinical signs of disease—such as mastitis, lameness, displaced abomasum, metritis, or digestive disorders or if their monitoring data were incomplete. Blood samples were collected once from each cow, and based on total-blood calcium (Ca) concentrations, the animals were divided into two groups [3,13,14]:

Group 1: cows with subclinical hypocalcemia (*n* = 20), (Ca < 2.0 mmol/L) mean of days in milk (DIM) 11.4 ± 5.0 DIM;

Group 2: healthy (*n* = 55) (Ca ≥ 2.0 mmol/L), mean of 10.7 ± 4.6 DIM.

On the same day as blood collection, data from the Lely automated milking system were retrieved for each cow. No additional oral or injectable calcium supplements (e.g., calcium boluses, drenches, or parenteral calcium preparations) were administered to any cows during the periparturient period, and no routine postpartum calcium supplementation protocol was implemented on the farm during the study.

### 2.4. Statistical Approach

All statistical analyses were performed using the KNIME Analytics Platform integrated within the same workflow. Descriptive statistics (mean, standard deviation, and sample size) were calculated for each variable within both groups. The normality of data distribution was assessed visually and through inspection of variance homogeneity. Because most variables displayed slight deviations from normality and unequal variances between groups, the Welch’s *t*-test was applied to compare group means. For robustness, non-parametric Mann–Whitney U tests were also computed, producing comparable results.

In addition to *p*-values, Cohen’s d effect sizes were calculated to quantify the magnitude of differences between groups (0.2 = small, 0.5 = medium, ≥0.8 = large). To account for multiple comparisons across numerous biomarkers, false discovery rate (FDR) adjustment was applied using the Benjamini–Hochberg procedure. The threshold for statistical significance was set at *p* < 0.05; parameters with 0.05 < *p* < 0.10 were considered trends, and significance after FDR correction is noted accordingly. To complement these descriptive comparisons, the results of the Benjamini–Hochberg correction for multiple testing are provided in Table 3, enabling a more robust interpretation of the observed between-group differences by controlling the false discovery rate.

To explore interrelationships among significant traits, Pearson correlation coefficients (r) were computed across all cows, integrating both blood biochemical and physiological variables The outcomes were visualized using a color-coded correlation heatmap. All statistical summaries and figures were exported automatically into Excel and image formats for further interpretation.

## 3. Results

Descriptive statistics for all investigated traits in hypocalcemic and healthy cows are presented in Table 2. The table includes mean values and standard deviations (mean ± SD) for each blood biochemical, milk composition, and behavioral parameter. Group 1 showed markedly lower serum Ca concentrations (*p* < 0.001), accompanied by reductions in P (*p* < 0.01) and Mg (*p* < 0.05). These differences confirm the expected alterations in mineral metabolism characteristic of hypocalcemia. To complement these descriptive comparisons, the results of the Benjamini–Hochberg correction for multiple testing are provided in Table 3, enabling a more robust interpretation of the observed between-group differences by controlling the false discovery rate.

Liver enzymes (AST, ALT, GGT) did not differ significantly between groups, and creatinine was significantly lower (*p* < 0.001) in hypocalcemic cows. Conversely, ALB, TP, GLUC, and Fe were lower (*p* < 0.05—0.001) in Group 1, reflecting reduced energy and protein metabolism and possible inflammatory responses.

Differences in milk composition were numerically evident but not statistically significant (*p* > 0.05): hypocalcemic cows tended to produce less milk and had higher fat and FPR, with slightly lower protein and lactose contents—suggesting reduced mammary efficiency and a transient negative energy balance.

Similarly, behavioral traits showed consistent, but non-significant tendencies: hypocalcemic cows displayed mildly increased rumination times and slightly lower milk temperatures (*p* ≈ 0.07—0.10).

**Figure 1:**
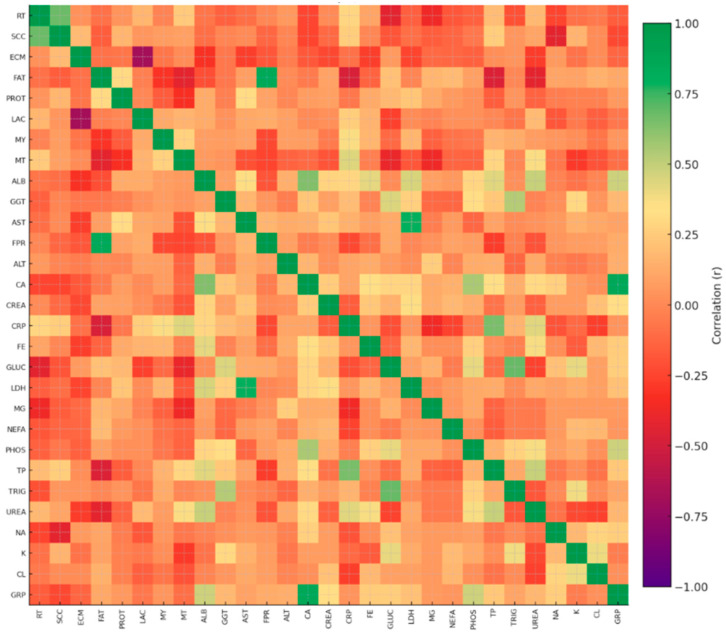
Correlation matrix of registered traits. Pearson correlation heatmap summarizing relationships among milk, behavioral, and serum biochemical variables measured in early-lactation dairy cows (n = 75; pairwise complete observations). Following parameters were measured: RT (min/day), SCC (×10^3^ cells/mL), ECM (mS/cm), FAT (%), PROT (%), LAC (%), MY (kg/day), MT (°C), ALB (g/L), AST (U/L), ALT (U/L), GGT (U/L), LDH (U/L), Ca (mmol/L), PHOS (mmol/L), Mg (mmol/L), NEFA (mmol/L), CREA (µmol/L), CRP (mg/L), Fe (µmol/L), TP (g/L), TRIG (mmol/L), UREA (mmol/L), Na (mmol/L), K (mmol/L), C (mmol/L), and group code (GRP) (unitless). Colors represent the strength and direction of correlations on a scale ranging from −1 to +1.

The correlation heatmap (Figure 1) indicated that most biochemical, milk, electrolyte, and behavioral variables exhibited weak linear associations, with the majority of absolute correlation coefficients ranging from 0.00 to 0.30. A limited number of moderate correlations were observed. The strongest association in the dataset was the positive correlation between Ca and ALB (r = 0.636, *p* ≈ 8.9 × 10^−10^). Ca also showed a weaker positive correlation with PHOS (r ≈ 0.34). Moderate correlations were present among selected protein-related markers, including ALB–TP (r ≈ 0.32) and ALB–Fe (r ≈ 0.35), as well as among liver enzymes such as AST–GGT (r ≈ 0.33) and AST–ALT (r ≈ 0.38). Milk variables (MF, MP, LAC, MY, ECM) displayed low correlations with biochemical traits, generally below r = 0.25. Electrolytes (Na, K, Cl), NEFA, CREA, and GLUC also showed low coefficients, typically within the 0.00 to 0.20 range. RT demonstrated minimal associations with serum variables, with correlations remaining below r = 0.15. The numerical table of correlations between the recorded parameters is provided in Table 4.

## 4. Discussion

This study demonstrates that subclinical hypocalcemia is accompanied by broad alterations in mineral balance, metabolic status, and physiological function, even in the absence of overt clinical disease. As expected, cows classified as hypocalcemic showed significantly reduced serum Ca concentrations, confirming their metabolic status, and these reductions were paralleled by lower PHOS and Mg values. These findings highlight the interconnected nature of mineral regulation in early lactation, where disturbances in calcium homeostasis commonly occur alongside imbalances in other macroelements. Such coordinated shifts reflect the underlying endocrine adjustments and the biological demands of the transition period, during which calcium mobilization, parathyroid hormone activity, and vitamin D–dependent absorption must rapidly increase to support lactation [4,15].

Beyond mineral alterations, significant reductions were observed in ALB, TP, GLUC, and Fe concentrations in healthy (group 2) cows. These findings point to broader systemic consequences of impaired calcium homeostasis. Lower ALB and TP may reflect reduced hepatic protein synthesis or altered nutrient partitioning during the early postpartum period [16,17]. A study that developed a liver tissue function index reported that total protein and albumin, among other indicators, are useful for monitoring metabolic dynamics during the peripartum period, with lower concentrations potentially reflecting altered hepatic function and nutrient metabolism at this stage [17]. The decline in glucose suggests an increased metabolic burden or reduced feed intake, both of which commonly accompany early-lactation disorders [18]. Moreover, the significantly lower serum glucose concentrations observed in hypocalcemic cows indicate that subclinical hypocalcemia may coincide with hypoglycemia or altered glucose metabolism during this critical period [19]. Similarly, reduced serum iron is consistent with either diminished dietary intake, impaired absorption, or redistribution of iron during inflammatory or metabolic stress [20]. The correlation analysis further supports this integrated physiological response. Serum Ca exhibited positive correlations with ALB and PHOS, reflecting their interconnected roles in mineral transport and homeostasis. Moderate associations with TP and Fe reinforce the involvement of protein-bound mineral fractions in calcium dynamics. In contrast, the weak associations for GLUC and CREA suggest that energy balance and renal indices are influenced by multiple physiological factors beyond Ca status alone. Collectively, these results indicate that subclinical hypocalcemia is associated with compromised energy and protein metabolism, even when clinical signs are absent.

In contrast to expectations, classical liver and kidney function indicators—AST, ALT, and GGT—did not differ significantly between hypocalcemic and healthy cows, and creatinine concentrations were significantly lower in the hypocalcemic group, suggesting that subclinical hypocalcemia did not impose marked hepatic or renal strain in this population. This interpretation is supported by findings from another study comparing clinical chemistry profiles of healthy and recumbent cows, which reported no significant differences in AST or GGT across various causes of recumbency, including hypocalcemia [21]. Together, these results indicate that hypocalcemia may not be consistently associated with alterations in AST or GGT beyond the typical risk period for parturient paresis. The lower CREA values in hypocalcemic cows may reflect reduced muscle turnover, lower metabolic rate, or decreased feed intake rather than impaired renal clearance [22].

Differences in milk composition were not statistically significant; however, cows in group 1 tended to produce slightly less milk, with marginally lower protein, lactose contents and milk temperature [6,23]. These tendencies align with the physiological expectation that cows experiencing early metabolic stress exhibit reduced mammary gland efficiency and increased reliance on lipid mobilization [24]. While the absence of statistical significance may reflect sample size or individual variability, the biological trends are consistent with known metabolic responses to calcium imbalance.

Similarly, behavioral traits showed non-significant but consistent patterns, with group 1 cows exhibiting slightly higher rumination times. This observation aligns with previous studies reporting that hypocalcemia does not affect rumination time [25]. However, some researchers believe that cows experiencing subclinical hypocalcemia tend to spend less time ruminating than their normocalcemic counterparts, suggesting that even mild reductions in calcium status may negatively influence normal rumination patterns during early lactation [26]. These subtle behavioral deviations highlight the value of continuous monitoring systems, which can detect early physiological shifts that may not yet be reflected in traditional clinical assessments [27].

Several limitations should be considered when interpreting the findings of this study. First, the sample size was relatively small, which may have limited the statistical power to detect differences in milk composition and behavioral parameters. Although biologically meaningful trends were observed, some non-significant outcomes may reflect insufficient sample numbers rather than a true absence of effect. Additionally, the study design was observational, preventing causal conclusions about the relationships between calcium status, metabolic indicators, and behavioral traits. Second, blood sampling was performed at a single time point, providing only a snapshot of biochemical status. Because calcium and related metabolic parameters can fluctuate markedly during early lactation, repeated sampling would have allowed a more detailed evaluation of temporal dynamics and individual variation. Importantly, only total calcium was measured, whereas ionized calcium—the biologically active fraction—was not assessed. Inclusion of ionized calcium would have offered a more accurate representation of calcium homeostasis and could have strengthened the classification of subclinical hypocalcemia. Similarly, although behavioral and milk parameters were continuously recorded, their interpretation was based on calcium status at only one sampling point, which may have limited the detection of short-term physiological responses to changing mineral balance. Environmental influences—such as housing conditions, temperature, and herd-management practices—were also not controlled and may have introduced variability. Lastly, sensor-derived behavioral data and in-line milk analyzer outputs may be subject to measurement error or device-specific bias, potentially affecting the precision of these variables. Despite these limitations, the integrative assessment of biochemical, behavioral, and milk composition traits provides meaningful insight into the multifactorial nature of subclinical hypocalcemia and underscores the potential of combined monitoring approaches in modern dairy herds.

## 5. Conclusions

This integrative evaluation demonstrates that subclinical hypocalcemia in early-lactation dairy cows is accompanied by coordinated disturbances in mineral balance, protein and energy metabolism, and subtle yet biologically meaningful changes in behavior and milk characteristics. Significant reductions in Ca, PHOS, Mg, ALB, TP, GLUC, and Fe underscore the systemic metabolic challenges associated with impaired calcium homeostasis, even in the absence of clinical signs. Although classical hepatic and renal indicators remained largely unchanged, lower CREA values likely reflect shifts in muscle turnover or metabolic rate, reinforcing the multifaceted physiological impact of subclinical hypocalcemia. Non-significant but consistent tendencies in milk composition align with known metabolic adaptations during early lactation, highlighting the sensitivity of these parameters to underlying mineral imbalances.

Despite limitations related to sample size and single-time point blood sampling, the combined use of biochemical markers, in-line milk analysis, and sensor-derived behavioral data proved valuable in characterizing the complexity of subclinical hypocalcemia. Future studies incorporating ionized calcium, longitudinal monitoring, and larger cohorts will be essential for refining diagnostic thresholds and improving predictive capabilities. Overall, this study supports the growing potential of precision-livestock monitoring systems to enhance the early detection and management of subclinical hypocalcemia, ultimately contributing to better health, welfare, and productivity in modern dairy herds.

## Figures and Tables

**Table 1:** Investigated milk, behavioral, and blood parameters with recording systems or analytical methods.

Parameter	Abbreviation	Measurement Source/System
Rumination time (min/d)	RT	Lely Astronaut^®^ A3 milking robot/Lely T4C management system
Milk yield (kg/d)	MY	
Milk fat (%)	F	
Milk protein (%)	P	
Milk lactose (%)	LAC	
Milk temperature °C	MT	
Fat-to-protein ratio	FPR	
Somatic cell count (10^3^ cells/mL)	SCC	
Milk electrical conductivity (Lely Astronaut conductivity score, unitless index)	ECM	
Albumin (g/L)	ALB	Hitachi 705 analyzer
Aspartate aminotransferase (U/L)	AST	
Gamma-glutamyltransferase (U/L)	GGT	
Alanine aminotransferase (U/L)	ALT	
Calcium (mmol/L)	Ca	
Creatinine (umol/L)	CREA	
C-reactive protein (mg/L)	CRP	
Iron (umol/L)	Fe	
Glucose (mmol/L)	GLUC	
Lactate dehydrogenase (U/L)	LDH	
Magnesium (mmol/L)	Mg	
Phosphorus (mmol/L)	PHOS	
Total protein (g/L)	TP	
Triglycerides (mmol/L)	TRIG	
Urea (mmol/L)	UREA	
Sodium (mmol/L)	Na	
Potassium (mmol/L)	K	
Chloride (mmol/L)	Cl	
Non-esterified fatty acids (mmol/L)	NEFA	Rx Daytona automated wet chemistry analyzer, Randox Laboratories Ltd.

**Table 2:** Investigated parameters based on calcium concentration groups.

Parameter	Mean Group 1 (n = 20)	SD	Mean Group 2 (n = 55)	SD	*p*_Value	Cohen_d
RT min/day	462.65	138.31	415.45	123.62	0.189	0.370
SCC 10^3^ cells/mL	382.90	405.79	215.47	281.10	0.101	0.526
ECM (Lely Astronaut conductivity score. unitless index)	71.85	5.58	70.49	4.13	0.328	0.299
F %	4.06	0.87	4.28	0.90	0.332	−0.253
P %	3.58	0.39	3.68	0.87	0.476	−0.135
LAC %	4.55	0.28	4.53	0.11	0.681	0.156
MY kg/d	36.05	14.37	38.48	24.26	0.320	−0.157
MT °C	39.40	0.88	39.20	0.94	0.403	0.214
ALB g/L ***	29.13	3.77	33.50	3.65	8.71 × 10^−5^	−1.186
GGT U/L	25.39	14.33	33.67	21.51	0.061	−0.417
AST U/L	80.36	37.42	88.17	33.56	0.418	−0.226
.FPR	1.13	0.21	1.20	0.22	0.270	−0.285
ALT U/L	22.71	6.66	29.34	25.09	0.077	−0.304
Ca mmol/L ***	1.86	0.10	2.35	0.14	9.24 × 10^−22^	−3.756
CREA umol/L ***	45.26	7.34	53.09	11.06	0.001	−0.767
CRP mg/L	9.19	4.65	9.70	5.23	0.683	−0.102
Fe umol/L *	15.63	5.64	19.72	7.27	0.014	−0.595
GLUC mmol/L **	2.86	0.65	3.46	1.09	0.005	−0.608
LDH U/L	1150.35	337.26	1321.96	362.25	0.064	−0.482
Mg mmol/L	1.33	1.06	1.51	1.22	0.552	−0.147
NEFA mmol/L	0.06	0.07	0.07	0.10	0.617	−0.109
PHOS mmol/L ***	1.63	0.38	2.09	0.39	5.45 × 10^−5^	−1.192
TP g/L *	64.64	9.80	70.04	9.91	0.043	−0.547
TRIG mmol/L	0.11	0.03	0.12	0.06	0.206	−0.238
UREA mmol/L	4.42	1.02	4.91	1.29	0.094	−0.400
Na mmol/L	127.05	28.16	136.49	7.57	0.154	−0.599
K mmol/L	4.33	0.49	4.29	0.36	0.771	0.089
Cl mmol/L	92.60	7.58	93.13	8.82	0.800	−0.062

* statistically significant at *p* < 0.05. ** statistically significant at *p* < 0.01. *** statistically significant at *p* < 0.001. Exact *p*-values and Cohen’s d values are provided to support interpretation.

**Table 3:** Benjamini–Hochberg statistical analysis.

Parameter	*p*_Value	Cohen_d	q_value
Ca (mmol/L) **	*p* > 0.001	−3.756	0.000
PHOS (mmol/L) **	*p* > 0.001	−1.192	0.001
ALB (g/L) **	*p* > 0.001	−1.186	0.001
CREA (umol/L) **	0.001	−0.767	0.006
GLUC (mmol/L) **	0.005	−0.608	0.028
Fe (µmol/L) *	0.014	−0.595	0.065
TP (g/L) *	0.043	−0.547	0.171
GGT (U/L)	0.061	−0.417	0.199
LDH (U/L)	0.064	−0.482	0.199
ALT (U/L)	0.077	−0.304	0.216
UREA (mmol/L)	0.094	−0.400	0.235
SCC (×10^3^ cells/mL)	0.101	0.526	0.235
Na (mmol/L)	0.154	−0.599	0.333
RT (min/day)	0.189	0.370	0.378
TRIG (mmol/L)	0.206	−0.238	0.385
FPR (ratio)	0.270	−0.285	0.472
MY (kg/day)	0.320	−0.157	0.490
ECM	0.328	0.299	0.490
Fat (%)	0.332	−0.253	0.490
Milk temperature (°C)	0.403	0.214	0.557
AST (U/L)	0.418	−0.226	0.557
Protein (%)	0.476	−0.135	0.606
Mg (mmol/L)	0.552	−0.147	0.671
NEFA (mmol/L)	0.617	−0.109	0.719
LAC (%)	0.681	0.156	0.735
CRP (mg/L)	0.683	−0.102	0.735
K (mmol/L)	0.771	0.089	0.799
Cl (mmol/L)	0.800	−0.062	0.800

* = *p* < 0.05; ** = significant after FDR correction (*q* < 0.05).

**Table 4:** Correalations between registrated parameters.

var1	var2	r
AST	ALT	0.175
AST	GGT	0.318
AST	LDH	0.790
AST	F %	0.134
AST	P %	0.233
AST	LAC %	0.102
AST	FPR	0.222
AST	MY	−0.062
AST	RT	0.016
ALT	GGT	0.014
ALT	LDH	0.079
ALT	F	0.032
ALT	P	−0.011
ALT	LAC	0.097
ALT	FPR	0.119
ALT	MY	0.908
ALT	RT	0.060
GGT	LDH	0.268
GGT	F	0.000
GGT	P	−0.071
GGT	LAC	−0.015
GGT	FPR	0.132
GGT	MY	−0.075
GGT	RT	0.047
LDH	F	0.017
LDH	P	0.164
LDH	LAC	0.027
LDH	FPR	0.026
LDH	MY	−0.096
LDH	RT	−0.025
F	P	0.287
F	LAC	−0.019
F	FPR	0.851
F	MY	0.082
F	RT	−0.145
P	LAC	0.007
P	FPR	0.035
P	MY	−0.006
P	RT	−0.027
LAC	FPR	0.101
LAC	MY	−0.019
LAC	RT	0.148
FPR	MY	0.085
FPR	RT	−0.029
MY	RT	−0.003

## Data Availability

The original contributions presented in the study are included in the article, further inquiries can be directed to the corresponding author.
